# Two Brothers with Atypical *UNC13D-*Related Hemophagocytic Lymphohistiocytosis Characterized by Massive Lung and Brain Involvement

**DOI:** 10.3389/fimmu.2017.01892

**Published:** 2017-12-21

**Authors:** Giuliana Giardino, Maia De Luca, Emilia Cirillo, Paolo Palma, Roberta Romano, Massimiliano Valeriani, Laura Papetti, Carol Saunders, Caterina Cancrini, Claudio Pignata

**Affiliations:** ^1^Department of Translational Medical Sciences, Federico II University of Naples, Naples, Italy; ^2^Unit of Immune and Infectious Diseases, University Department of Pediatrics (DPUO), Bambino Gesù Children’s Hospital, Rome, Italy; ^3^Research Unit in Congenital and Perinatal Infection, Unit of Immune and Infectious Diseases, University Department of Pediatrics (DPUO), Bambino Gesù Children’s Hospital, Rome, Italy; ^4^Neurology Unit, Bambino Gesù Children’s Hospital, Rome, Italy; ^5^Center for Pediatric Genomic Medicine, Children’s Mercy-Kansas City, Kansas City, MO, United States; ^6^School of Medicine, University of Missouri-Kansas City, Kansas City, MO, United States; ^7^Department of Pathology, Children’s Mercy-Kansas City, Kansas City, MO, United States; ^8^Department of Systems Medicine, University of Rome Tor Vergata, Rome, Italy

**Keywords:** *UNC13D*, FHL3, atypical FHL, CNS-HLH, extracorporeal membrane oxygenation

## Abstract

Hemophagocytic lymphohistiocytosis (HLH) is a potentially fatal hyperinflammatory condition. Variants in different genes have been associated with the familial forms of the syndrome (FHL), usually presenting within the first 2 years of life. Due to increasing awareness of the signs and symptoms of HLH and a better understanding of the genetic basis of the disease, FHL has been increasingly diagnosed in patients presenting beyond infancy. Here, we report on two brothers with atypical, late-onset HLH in which whole exome sequencing revealed a homozygous pathogenic *UNC13D* variant. In the first brother, the clinical phenotype was dominated by a massive lung involvement. In the second brother a progressive neurological deterioration was observed. In both cases, the clinical manifestations at symptom onset were misleading, making the diagnosis difficult to achieve. This report expands the spectrum of clinical presentations of FLH3. Moreover, it highlights the importance to warn clinicians to keep a high level of suspicion in patients presenting with fever, cytopenia, splenomegaly of unknown origin, and unresponsiveness to conventional treatment even beyond early childhood. Moreover, this report emphasizes that insidious neurologic symptoms may represent the initial or sole presenting sign of FHL, even in the absence of peripheral signs of activation.

## Introduction

Hemophagocytic lymphohistiocytosis (HLH) is a potentially fatal hyperinflammatory condition characterized by prolonged and unexplained fever, unresponsiveness to conventional treatment, hepatosplenomegaly, cytopenia, hypertriglyceridemia, and hypofibrinogenemia (1, 2). Variants in four different genes have been associated with the familial forms of the syndrome (FHL). FHL-2 is due to variations in the *PRF1* gene, encoding perforin 1, implicated in target cell lysis (3). *UNC13D* encoding MUNC13-4, *STX11* encoding SYNTAXIN11, and *STXBP2* encoding MUNC18-2 are implicated in the trafficking and exocytosis of lytic granules, and mediate the release at the cell surface of perforin1 and other effector molecules implicated in cytotoxicity (4–7). The impairment of the cytotoxic activity may lead to uncontrolled activation of cytotoxic T cells and macrophages, which finally result in a hyperinflammation, T-cell, and macrophage infiltration of various organs including bone marrow, liver, and the central nervous system (8).

FHL usually presents within the first 2 years of life (9). However, the increasing awareness of the signs and symptoms of HLH and the better comprehension of the genetic basis of the disease allow the identification of FHL in patients presenting beyond infancy and sometimes before the development of the HLH (10). Atypical presentations have been reported in adolescents and even in adults, characterized by milder and often recurrent HLH episodes and prolonged survival in the absence of hematopoietic stem cell transplantation (HSCT), unusual in patients with the typical disease. Here, we report on two brothers with atypical, late-onset HLH in which whole exome sequencing (WES) revealed a homozygous pathogenic *UNC13D* variant. In both cases, the clinical manifestations at symptom onset were misleading, making the diagnosis difficult to achieve.

## Case Report

**Figure 1:**
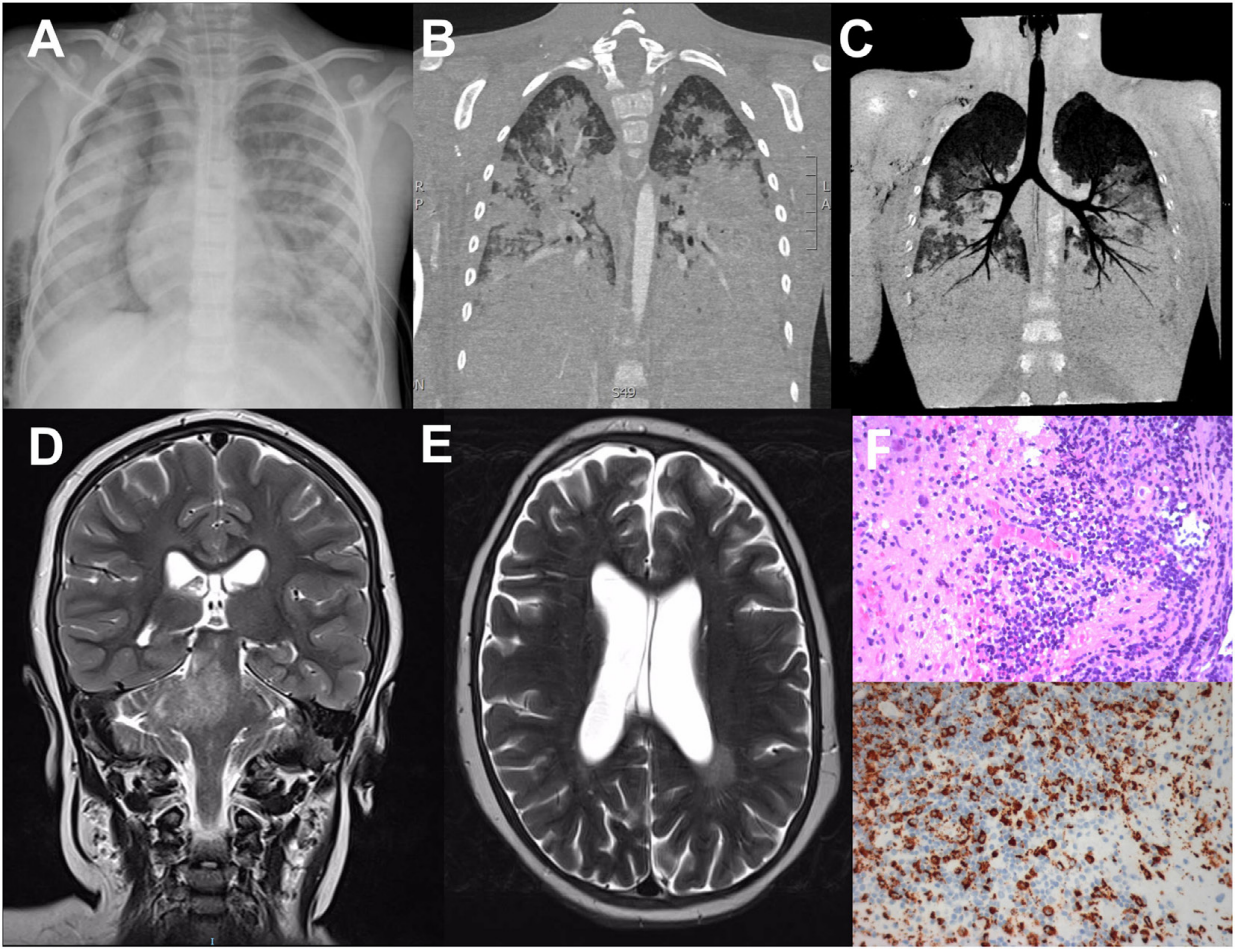
Radiological findings in the dizygotic twins homozygous for 1847A>G (p.Glu616Gly) in *UNC13D*. **(A)** Thoracic Rx of the patient 1; broad area of opacity involving the right mediobasal lobe and the left basal lobe, with bilateral hilar and perihilar infiltration. **(B,C)** Pulmonary CT scan of the patient 1; massive bilateral pulmonary consolidations with alveolar involvement, only sparing the apical lobes. **(D,E)** Brain MRI scan of the patient 2; T2w image with hyperintense signal in the left periventricular region, in pons, right cerebellar peduncles, and right cerebellar hemisphere. **(F)** Hematoxylin-eosin staining of the brain biopsy showing lymphohistiocytic perivascular and intraparenchymal inflammatory infiltrate (upper panel); immunohistochemical staining for the histiocytic marker CD68 (lower panel).

Written informed consent was obtained from the parents for the publication of both the cases. The patients were dizygotic twins born preterm (33 weeks) following conception by *in vitro* fertilization, yielding a triplet pregnancy complicated by *in utero* death of the third fetus. Parents were not consanguineous and familial history was unremarkable, apart from a history of recurrent abortions in the mother. At the age of 6.2 years, after an uneventful childhood, the first brother developed fever resistant to antibiotics and severe, rapidly progressive dyspnea, requiring the admission to a pediatric intensive care unit for mechanical ventilation. The rapid progression of such a severe lung involvement raised the suspicion of a primary immunodeficiency (11). Clinical examination revealed splenomegaly and macular truncal rash. Laboratory examinations revealed trilineage cytopenia, hypertriglyceridemia, hypofibrinogenemia, and hyperferritinemia (Table 1). The analysis of the bone marrow aspirate revealed hypocellularity, but lymphocytes and histiocytes infiltration and hemophagocytosis were not observed. Lung Rx and CT scan revealed massive bilateral pulmonary consolidations with alveolar involvement, only sparing the apical lobes (Figures 1A–C). *Klebsiella pneumoniae* and *Candida albicans* were isolated from the bronchial aspirate while the pleural fluid culture was negative. Nasal, pharyngeal and rectal swab, urine culture, mannan, galactomannan and PCR for *HSV1/2, Parvovirus B19, HHV8, HHV6, CMV, EBV*, and other respiratory viruses in the blood and bronchial aspirate were negative. Cytological examination of the pleural fluid showed an increase of the protein levels and of the leukocytes, in particular lymphocytes. Basal immunological examination revealed hypogammaglobulinemia (IgG 315 mg/dL, IgA 46 mg/dL, IgM 58 mg/dL) with normal production of specific antibodies and a slight increase of IgE levels (249 IU/mL). A marked lymphopenia was observed, despite normal T, B, and NK cells percentages, apart from an increase of the CD3+ HLADR+ cells (Table 1). WES was performed due to suspicion of a CID with secondary HLH. Treatment with intravenous immunoglobulins, antibiotics, and antifungals was started. Venous extracorporeal membrane oxygenation (ECMO) was initiated due to the development of bilateral massive pneumothorax, but unfortunately the patient died of cardiopulmonary collapse.

**Table 1:** Comparison of clinical and laboratory markers in the two twins carrying the same UNC13D mutation.

	Patient 1	Patient 2
*UNC13D* mutation	Homozygous 1847A>G	Homozygous 1847A>G
Age at onset	6.2 years	3 years
Symptoms at the onset	Fever and severe dyspnea	Pervasive developmental disorder
Fever	Yes	No
Splenomegaly	Yes	Yes
CNS involvement	No	Yes
Lung involvement	Massive bilateral consolidations	Signs of pulmonary hypertension
WBC (cells/mm^3^)	800	7,750
Neutrophil count (cells/mm^3^)	430	5,450
Lymphocyte count (cells/mm^3^)	320	1,880
Platelet count (cells/mm^3^)	27,000	241,000
Hemoglobin levels (g/dL)	7.8	14.9
Fibrinogen (mg/dL)	89	n.a.
Triglycerides (mg/dL)	1,556	110
Ferritin (ng/mL)	2,550	84
Albumin (gr/dL)	2.4	5
IgG/IgA/IgM (mg/dL)	315/46/58	487/45/120
IgE (IU/mL)	249	n.a.
CD3+ % (cells/mm^3^)	57% (182)	77.5%
CD4+ % (cells/mm^3^)	34% (109)	33%
CD8+ % (cells/mm^3^)	13% (42)	33.2%
CD19+ % (cells/mm^3^)	8% (26)	21%
CD16+ CD56+ % (cells/mm^3^)	22% (70)	1.5%
CD4+ CD45RA+ % (cells/mm^3^)	n.a.	6.6%
CD4+ CD45RO+ % (cells/mm^3^)	n.a.	26.4%
CD8+ CD45RA+	n.a.	14.9%
CD8+ CD45RO+	n.a.	18.3%
CD3+ HLADR+	27%	n.a.
Granule release assay	n.a.	Absent degranulation
Perforin expression	n.a.	Normal
Bone marrow aspirate	Hypocellularity	Lymphohistiocytic infiltration

*CNS, central nervous system; WBC, white blood cells; n.a., not available*.

The second brother developed a pervasive developmental disorder at the age of 3 years. By age 5, his neurological condition deteriorated with the development of asthenia, ataxia, and nystagmus, facilitating a neurological evaluation. Brain MRI revealed areas of demyelination enhanced by contrast agent, involving pons, cerebellar peduncles, cerebellum, and white matter of the left temporal lobe (Figures 1D,E). CSF evaluation revealed pleocytosis and oligoclonal bands. Based on the radiological findings and favorable response to steroid treatment, a diagnosis of Chronic Lymphocytic Inflammation with Pontine Perivascular Enhancement Responsive to Steroids was suspected and the patient was started on Azathioprine. However, clinical and radiological findings deteriorated despite the immunosuppressive treatment and a brain biopsy was performed showing a lymphohistiocytic perivascular and intraparenchymal inflammatory infiltrate (Figure 1F). A CNS-HLH was suspected. Peripheral markers of activation were evaluated and found to be normal (Table 1). Splenomegaly and bone marrow lymphohistiocytic infiltration were detected. Immunological studies revealed normal IgG, IgA, and IgM levels and slightly increased CD3+ cells, mainly showing a memory phenotype, absent degranulation of the NK cells after the stimulus with K562 cells, normal perforin expression, suggesting a diagnosis of HLH (Table 1).

In the first brother, WES revealed a homozygous pathogenic *UNC13D* variant, 1847A>G (p.Glu616Gly). This variant has been previously reported in affected individuals, and functionally demonstrated to result in deranged splicing of the gene (12, 13). It is absent from population databases. The second twin was genotyped for the variant by Sanger sequencing, revealing homozygosity for the same *UNC13D* variant. The parents were both heterozygous. A diagnosis of isolated CNS-HLH was made, and the patient was started on the HLH-2004 protocol. The patient unfortunately died of transplant-related complications.

## Discussion

Hemophagocytic lymphohistiocytosis represents the clinical and immunological manifestation of several diseases including FHL, characterized by a well-known pathogenic mechanism and a large number of disorders in which pathogenesis is still poorly understood. Indeed, HLH has been found in association with inborn errors of the metabolism (14–16), autoimmune disease (17), congenital immunodeficiencies, such as chronic granulomatous disease (18), DiGeorge syndrome (19), severe combined immunodeficiency (20), and X-linked agammaglobulinemia (21, 22), and other disorders (23–25). These last cases, described as “secondary” HLH, usually present in older children. More recently, the increasing awareness of the presenting signs of the disease and the development of new tools for the diagnosis of these conditions have made possible the identification also in patients presenting later in life with atypical symptoms (26). Considering the similarities between atypical FHL and secondary HLH, it can be difficult in some cases to achieve a rapid diagnosis. Moreover, in many cases of atypical FHL, the peripheral signs of HLH may be completely absent, making the diagnosis even more difficult to achieve. In a recent paper, Gray et al reported on a case of late-onset FHL3 in which the clinical presentation was characterized by growth arrest, inflammatory arachnoiditis, and dysgammaglobulinemia, without any signs of HLH (10). Similarly, the hypomorphic A91V variation has been recently described in association with atypical late-onset HLH in a patient with neuromyelitis optica (27).

In this report, we describe two male dizygotic twins with atypical FHL3 (12, 13, 28). In both patients, the onset was delayed compared to the median age of 4.1 months reported in a wide cohort of FHL3 patients (13). Missense variants in *UNC13D* are associated with a later onset as compared with loss of function variants and median age at the diagnosis was 117.5 months in four patients with homozygous c.1847A>G (13). The two brothers presented with very different clinical manifestations. In the first brother the clinical picture was dominated by a massive lung involvement and laboratory features suggested a secondary HLH in the context of a CID (24). WES failed to detect mutations other than *UNC13D*. However, even though primary immunodeficiencies have been traditionally considered as monogenic disorders, in the recent years high throughput sequencing techniques have highlighted that atypical phenotypes may be explained by the coexistence of multiple mutations (29–33). Since WES may not be able to detect large deletion/insertion and deep intronic mutations, we cannot exclude that a second mutation may have had a role in shaping such a complex clinical phenotype. On the other hand, even though immunological alterations are not usually found in patients with typical FHL, recent evidence suggests that different immunological alterations including antibody deficiency, low B-cell numbers, reduced percentage of marginal-zone like and class-switched memory B cells, and susceptibility to bacterial sinopulmonary infections may be detected in patients with different forms of FHL (34). In some cases, hypogammaglobulinemia and defects of specific antibodies developed after recurrent mild episodes of HLH (34). Interestingly, the immunological alterations are not usually found in patients with typical FHL, suggesting that in atypical forms a silent chronic T cell activation may impair the B cell development leading to the development of hypogammaglobulinemia (34). For this reason, the association of a clinical picture of common variable immunodeficiency/CID, with splenomegaly, cytopenia, and episodes of unexplained fever should address the diagnosis also toward atypical FHL (34).

Lung involvement is not well characterized in HLH, especially in pediatric patients and guidelines on the treatment of these conditions are also lacking (35). In a cohort of adult patients, lung involvement, due to lymphocytic infiltration or infection, was reported in about 54% of cases and associated with a worse prognosis (36). However, adult HLH may have a different pathogenesis compared to children affected with FHL. In the patient described, *Klebsiella* pneumoniae and *Candida* albicans may have played a role in triggering the lung involvement and, moreover, the hypogammaglobulinemia along with the marked lymphopenia may have had a role in the rapid deterioration of the clinical conditions. However, it should be noted that five out of eight HLH diagnostic criteria were present at the diagnosis (fever, splenomegalia, trilineage cytopenia, hyperferritinemia, hypofibrinogenemia/hypertriglyceridemia), suggesting that HLH also had a role in shaping the clinical picture. Moreover, the patient did not show any sign of improvement despite 3 months of intravenous treatment with immunoglobulins, different antifungals, and antibiotics, suggesting an inflammatory more than infectious condition. Even though lung biopsy was not performed in our patients, all cultures, including pleural fluid were negative, with pleural fluid showing increased levels of proteins and lymphocytes, suggesting lung infiltration by activated lymphocytes and histiocytes. In our patient, ECMO was initiated due to the development of bilateral massive pneumothorax. However, in a recent study on a small cohort of patients, survival of pediatric HLH patients on ECMO was worse than patients without HLH on ECMO (37). It should be noted that the severity of the clinical manifestations and the high mortality related to the lung involvement may themselves lead to a reduced estimation of the incidence of the lung involvement in FHL, since the patient may die without receiving a genetic diagnosis.

The second brother presented at the age of 3 years with signs of a pervasive developmental disorder. His neurological conditions deteriorated progressively over a very long period. While lung infiltration is an unusual presentation of FHL (12, 13), neurologic signs are present in about 2/3 of the patients with FLH at the onset and may also develop later in the course of the disease (38, 39). Common neurological manifestations include irritability, seizures, bulging fontanel in infants, neck stiffness, hypotonia, and hypertonia (9, 40, 41). Other less common signs include nonspecific signs of increased intracranial pressure, cranial nerve palsy, ataxia, hemiplegia, quadriplegia, blindness, and coma (9, 40, 41). CSF examination usually shows elevated protein, increased number of mononuclear cells, and occasionally hemophagocytosis (9, 40, 41). Typical radiologic findings include demyelination or impaired myelination, gray and white matter multifocal inflammation mimicking neuroinflammatory disorders including multiple sclerosis and acute disseminated encephalomyelitis (41, 42). FHL3 patients seem to be even more prone to develop CNS involvement. In a recent study, 60% of the patients with FHL3 showed a CNS involvement compared with the 32% in patients with FHL2 (13). Moreover, the variant identified in these twins has been frequently associated with CNS involvement (13). Of note, in our patient, more typical clinical neurological manifestations associated with HLH, including demyelination, ataxia, and cerebral palsy (43), were preceded by a pervasive developmental disorder, suggesting a very slow and insidious process which delayed the diagnosis. Moreover, the patient never showed any sign of systemic HLH. Previous evidence suggest that primary HLH may also represent the underlying cause of unknown CNS inflammation even in the absence of overt clinical signs of HLH, suggesting that FHL should be included in the differential diagnosis of unclassified progressive neuroinflammatory disease (44–50). T and NK cell degranulation assays and the evaluation of the expression of intracellular perforin can be used for the prompt identification of such patients, allowing a targeted treatment (51). Corticosteroids, etoposide, cyclosporine A, commonly used for the treatment of systemic HLH, along with intrathecal methotrexate and corticosteroids represent the standard, first-line therapeutic approach also for the treatment of CNS-HLH (1, 9, 38). Antithymocyte globulin has also been used, in association with steroids, cyclosporin A, and intrathecal methotrexate injection to achieve remission in HLH (52). However, the efficacy of this association on CNS is comparable to the HLH-94 protocol (38, 52). HSCT also represents an important tool for the treatment of CNS-HLH especially in patients with familial forms, or with severe, persistent or reactivated HLH (38). Recently, novel therapeutic approaches are emerging as effective in the treatment of the refractory and complicated HLH even though many of these are still considered as second line or salvage experimental treatments. Among these, alemtuzumab has shown promising results in the treatment of the refractory HLH even though its effectiveness in the treatment of refractory CNS-HLH has not been clarified yet (53). Based on encouraging preclinical data from animal model, a clinical trial (NCT01818492) is currently ongoing to evaluate the efficacy of anti-IFNγ monoclonal antibody NI-0501 in the treatment of refractory HLH. Finally, the JAK1/2 inhibitor ruxolitinib has been recently shown to be effective in reducing the CNS involvement in a Rab27a^−/−^ mouse model (54).

## Concluding Remarks

In conclusion, this report expands the spectrum of clinical presentations of FLH3. It is important to warn clinicians to keep a high level of suspicion in patients presenting with fever, cytopenia, splenomegaly of unknown origin, and unresponsiveness to conventional treatment even beyond early childhood. Moreover, it must be emphasized that insidious neurologic symptoms may represent the initial or sole presenting sign of FHL, even in the absence of any overt clinical manifestations of HLH. Considering that in FHL3 diagnostic delay may be fatal or result in irreversible neurologic sequelae, clinicians should be aware that unclassified neuroinflammatory diseases could be the sole initial sign of FHL. In this context, next-generation sequencing techniques and flow cytometric assays may play an important role in addressing the diagnosis. The precocious diagnosis of these conditions is pivotal to establish the best therapeutic approach.

## Ethics Statement

The study was carried out in accordance with the Declaration of Helsinki and was approved by the institutional ethics committee of the University Federico II of Naples. Informed consent was obtained from all patients before the study.

## Author Contributions

CP, CC, GG, ML, and EC organized, collected, and analyzed the data and wrote the manuscript. PP, RR, MV, and LP followed up the patients, collected the data, and performed the experiments. CS performed whole exome sequencing; all authors reviewed and approved the manuscript.

## Conflict of Interest Statement

The authors declare that the research was conducted in the absence of any commercial or financial relationships that could be construed as a potential conflict of interest.

## References

[1] Henter JI Horne A Arico M Egeler RM Filipovich AH Imashuku S HLH-2004: diagnostic and therapeutic guidelines for hemophagocytic lymphohistiocytosis. Pediatr Blood Cancer (2007) 48(2):124–31. 10.1002/pbc.21039 16937360

[2] Giardino G Veropalumbo C Ruggiero G Naddei R Rubino V Udhayachandran A Phenotypic characterization and outcome of paediatric patients affected with haemophagocytic syndrome of unknown genetic cause. Br J Haematol (2013) 162(5):713–7. 10.1111/bjh.12421 23808825

[3] Stepp SE Dufourcq-Lagelouse R Deist FL Bhawan S Certain S Mathew PA Perforin gene defects in familial hemophagocytic lymphohistiocytosis. Science (1999) 286(5446):1957–9. 10.1126/science.286.5446.1957 10583959

[4] zur Stadt U Schmidt S Kasper B Beutel K Diler AS Henter JI Linkage of familial hemophagocytic lymphohistiocytosis (FHL) type-4 to chromosome 6q24 and identification of mutations in syntaxin 11. Hum Mol Genet (2005) 14(6):827–34. 10.1093/hmg/ddi076 15703195

[5] zur Stadt U Rohr J Seifert W Koch F Grieve S Pagel J Familial hemophagocytic lymphohistiocytosis type 5 (FHL-5) is caused by mutations in Munc18-2 and impaired binding to syntaxin 11. Am J Hum Genet (2009) 85(4):482–92. 10.1016/j.ajhg.2009.09.005 19804848 PMC2756548

[6] Cote M Menager MM Burgess A Mahlaoui N Picard C Schaffner C Munc18-2 deficiency causes familial hemophagocytic lymphohistiocytosis type 5 and impairs cytotoxic granule exocytosis in patient NK cells. J Clin Invest (2009) 119(12):3765–73. 10.1172/jci40732 19884660 PMC2786810

[7] Feldmann J Callebaut I Raposo G Certain S Bacq D Dumont C Munc13-4 is essential for cytolytic granules fusion and is mutated in a form of familial hemophagocytic lymphohistiocytosis (FHL3). Cell (2003) 115(4):461–73. 10.1016/S0092-8674(03)00855-9 14622600

[8] Fischer A Latour S de Saint Basile G. Genetic defects affecting lymphocyte cytotoxicity. Curr Opin Immunol (2007) 19(3):348–53. 10.1016/j.coi.2007.04.006 17433652

[9] Madkaikar M Shabrish S Desai M. Current updates on classification, diagnosis and treatment of hemophagocytic lymphohistiocytosis (HLH). Indian J Pediatr (2016) 83(5):434–43. 10.1007/s12098-016-2037-y 26872683

[10] Gray PE Shadur B Russell S Mitchell R Buckley M Gallagher K Late-onset non-HLH presentations of growth arrest, inflammatory arachnoiditis, and severe infectious mononucleosis, in siblings with hypomorphic defects in UNC13D. Front Immunol (2017) 8:944. 10.3389/fimmu.2017.00944 28848550 PMC5552658

[11] Cirillo E Giardino G Gallo V D’Assante R Grasso F Romano R Severe combined immunodeficiency – an update. Ann N Y Acad Sci (2015) 1356:90–106. 10.1111/nyas.12849 26235889

[12] Santoro A Cannella S Bossi G Gallo F Trizzino A Pende D Novel Munc13-4 mutations in children and young adult patients with haemophagocytic lymphohistiocytosis. J Med Genet (2006) 43(12):953–60. 10.1136/jmg.2006.041863 16825436 PMC2563207

[13] Sieni E Cetica V Santoro A Beutel K Mastrodicasa E Meeths M Genotype-phenotype study of familial haemophagocytic lymphohistiocytosis type 3. J Med Genet (2011) 48(5):343–52. 10.1136/jmg.2010.085456 21248318 PMC4115201

[14] Henkes M Finke J Warnatz K Ammann S Stadt UZ Janka G Late-onset hemophagocytic lymphohistiocytosis (HLH) in an adult female with Griscelli syndrome type 2 (GS2). Ann Hematol (2015) 94(6):1057–60. 10.1007/s00277-014-2284-9 25544030

[15] Kardas F Patiroglu T Unal E Chiang SC Bryceson YT Kendirci M. Hemophagocytic syndrome in a 4-month-old infant with biotinidase deficiency. Pediatr Blood Cancer (2012) 59(1):191–3. 10.1002/pbc.23247 22605457

[16] Sharpe LR Ancliff P Amrolia P Gilmour KC Vellodi A. Type II Gaucher disease manifesting as haemophagocytic lymphohistiocytosis. J Inherit Metab Dis (2009) 32(Suppl 1):S107–10. 10.1007/s10545-009-1091-2 19267217

[17] Ravelli A Minoia F Davi S Horne A Bovis F Pistorio A 2016 classification criteria for macrophage activation syndrome complicating systemic juvenile idiopathic arthritis: a European league against rheumatism/American College of Rheumatology/Paediatric Rheumatology International Trials Organisation collaborative initiative. Arthritis Rheumatol (2016) 68(3):566–76. 10.1002/art.39332 26314788

[18] Valentine G Thomas TA Nguyen T Lai YC. Chronic granulomatous disease presenting as hemophagocytic lymphohistiocytosis: a case report. Pediatrics (2014) 134(6):e1727–30. 10.1542/peds.2014-2175 25422023

[19] Arico M Bettinelli A Maccario R Clementi R Bossi G Danesino C. Hemophagocytic lymphohistiocytosis in a patient with deletion of 22q11.2. Am J Med Genet (1999) 87(4):329–30. 10.1002/(SICI)1096-8628(19991203)87:4<329::AID-AJMG9>3.0.CO;2-M 10588839

[20] Patiroglu T Haluk Akar H van den Burg M Unal E Akyildiz BN Tekerek NU X-linked severe combined immunodeficiency due to a novel mutation complicated with hemophagocytic lymphohistiocytosis and presented with invagination: a case report. Eur J Microbiol Immunol (Bp) (2014) 4(3):174–6. 10.1556/eujmi-d-14-00019 25215194 PMC4160797

[21] Ozturk C Sutcuoglu S Atabay B Berdeli A. X-linked agammaglobulinemia presenting with secondary hemophagocytic syndrome: a case report. Case Rep Med (2013) 2013:742795. 10.1155/2013/742795 23424595 PMC3568855

[22] Schultz KA Neglia JP Smith AR Ochs HD Torgerson TR Kumar A. Familial hemophagocytic lymphohistiocytosis in two brothers with X-linked agammaglobulinemia. Pediatr Blood Cancer (2008) 51(2):293–5. 10.1002/pbc.21573 18421721

[23] Pasic S Micic D Kuzmanovic M. Epstein-Barr virus-associated haemophagocytic lymphohistiocytosis in Wiskott-Aldrich syndrome. Acta Paediatr (2003) 92(7):859–61. 10.1111/j.1651-2227.2003.tb02548.x 12892170

[24] Bode SF Ammann S Al-Herz W Bataneant M Dvorak CC Gehring S The syndrome of hemophagocytic lymphohistiocytosis in primary immunodeficiencies: implications for differential diagnosis and pathogenesis. Haematologica (2015) 100(7):978–88. 10.3324/haematol.2014.121608 26022711 PMC4486233

[25] Ishii E. Hemophagocytic lymphohistiocytosis in children: pathogenesis and treatment. Front Pediatr (2016) 4:47. 10.3389/fped.2016.00047 27242976 PMC4865497

[26] Sieni E Cetica V Piccin A Gherlinzoni F Sasso FC Rabusin M Familial hemophagocytic lymphohistiocytosis may present during adulthood: clinical and genetic features of a small series. PLoS One (2012) 7(9):e44649. 10.1371/journal.pone.0044649 22970278 PMC3436758

[27] Palterer B Brugnolo F Sieni E Barilaro A Parronchi P. Neuromyelitis optica, atypical hemophagocytic lymphohistiocytosis and heterozygous perforin A91V mutation. J Neuroimmunol (2017) 311:10–3. 10.1016/j.jneuroim.2017.08.003 28863861

[28] Picard C Al-Herz W Bousfiha A Casanova JL Chatila T Conley ME Primary immunodeficiency diseases: an update on the classification from the international union of immunological societies expert committee for primary immunodeficiency 2015. J Clin Immunol (2015) 35(8):696–726. 10.1007/s10875-015-0201-1 26482257 PMC4659841

[29] Massaad MJ Zhou J Tsuchimoto D Chou J Jabara H Janssen E Deficiency of base excision repair enzyme NEIL3 drives increased predisposition to autoimmunity. J Clin Invest (2016) 126(11):4219–36. 10.1172/jci85647 27760045 PMC5096910

[30] Tangye SG Genetic cause of immune dysregulation – one gene or two? J Clin Invest (2016) 126(11):4065–7. 10.1172/jci90831 27760052 PMC5096897

[31] Casanova JL Fieschi C Zhang SY Abel L. Revisiting human primary immunodeficiencies. J Intern Med (2008) 264(2):115–27. 10.1111/j.1365-2796.2008.01971.x 18544117

[32] Gallo V Dotta L Giardino G Cirillo E Lougaris V D’Assante R Diagnostics of primary immunodeficiencies through next-generation sequencing. Front Immunol (2016) 7:466. 10.3389/fimmu.2016.00466 27872624 PMC5098274

[33] Hoyos-Bachiloglu R Chou J Sodroski CN Beano A Bainter W Angelova M A digenic human immunodeficiency characterized by IFNAR1 and IFNGR2 mutations. J Clin Invest (2017) 127:12. 10.1172/JCI93486 29106381 PMC5707159

[34] Rohr J Beutel K Maul-Pavicic A Vraetz T Thiel J Warnatz K Atypical familial hemophagocytic lymphohistiocytosis due to mutations in UNC13D and STXBP2 overlaps with primary immunodeficiency diseases. Haematologica (2010) 95(12):2080–7. 10.3324/haematol.2010.029389 20823128 PMC2995566

[35] Jordan MB Allen CE Weitzman S Filipovich AH McClain KL. How I treat hemophagocytic lymphohistiocytosis. Blood (2011) 118(15):4041–52. 10.1182/blood-2011-03-278127 21828139 PMC3204727

[36] Seguin A Galicier L Boutboul D Lemiale V Azoulay E Pulmonary involvement in patients with hemophagocytic lymphohistiocytosis. Chest (2016) 149(5):1294–301. 10.1016/j.chest.2015.11.004 26836913

[37] Cashen K Chu RL Klein J Rycus PT Costello JM. Extracorporeal membrane oxygenation outcomes in children with hemophagocytic lymphohistiocytosis. Perfusion (2017) 32(2):151–6. 10.1177/0267659116667804 27625333

[38] Trottestam H Horne A Arico M Egeler RM Filipovich AH Gadner H Chemoimmunotherapy for hemophagocytic lymphohistiocytosis: long-term results of the HLH-94 treatment protocol. Blood (2011) 118(17):4577–84. 10.1182/blood-2011-06-356261 21900192 PMC3208276

[39] Kim MM Yum MS Choi HW Ko TS Im HJ Seo JJ Central nervous system (CNS) involvement is a critical prognostic factor for hemophagocytic lymphohistiocytosis. Korean J Hematol (2012) 47(4):273–80. 10.5045/kjh.2012.47.4.273 23320006 PMC3538799

[40] Haddad E Sulis ML Jabado N Blanche S Fischer A Tardieu M. Frequency and severity of central nervous system lesions in hemophagocytic lymphohistiocytosis. Blood (1997) 89(3):794–800. 9028310

[41] Zhang K Filipovich AH Johnson J Marsh RA Villanueva J Hemophagocytic lymphohistiocytosis, familial. GeneReviews. Seattle: University of Washington (1993). Available from: https://www.ncbi.nlm.nih.gov/pubmed/20301617

[42] Weisfeld-Adams JD Frank Y Havalad V Hojsak JM Posada R Kaicker SM Diagnostic challenges in a child with familial hemophagocytic lymphohistiocytosis type 3 (FHLH3) presenting with fulminant neurological disease. Childs Nerv Syst (2009) 25(2):153–9. 10.1007/s00381-008-0744-z 19023578

[43] Deiva K Mahlaoui N Beaudonnet F de Saint Basile G Caridade G Moshous D CNS involvement at the onset of primary hemophagocytic lymphohistiocytosis. Neurology (2012) 78(15):1150–6. 10.1212/WNL.0b013e31824f800a 22422896

[44] Feldmann J Menasche G Callebaut I Minard-Colin V Bader-Meunier B Le Clainche L Severe and progressive encephalitis as a presenting manifestation of a novel missense perforin mutation and impaired cytolytic activity. Blood (2005) 105(7):2658–63. 10.1182/blood-2004-09-3590 15598808

[45] Shinoda J Murase S Takenaka K Sakai N. Isolated central nervous system hemophagocytic lymphohistiocytosis: case report. Neurosurgery (2005) 56(1):187. 10.1227/01.NEU.0000146210.13318.E8 15617602

[46] Moshous D Feyen O Lankisch P Schwarz K Schaper J Schneider M Primary necrotizing lymphocytic central nervous system vasculitis due to perforin deficiency in a four-year-old girl. Arthritis Rheum (2007) 56(3):995–9. 10.1002/art.22442 17328077

[47] Rostasy K Kolb R Pohl D Mueller H Fels C Moers AV CNS disease as the main manifestation of hemophagocytic lymphohistiocytosis in two children. Neuropediatrics (2004) 35(1):45–9. 10.1055/s-2004-815791 15002052

[48] Henter JI Elinder G. Cerebromeningeal haemophagocytic lymphohistiocytosis. Lancet (1992) 339(8785):104–7. 10.1016/0140-6736(92)91008-V 1345827

[49] Kieslich M Vecchi M Driever PH Laverda AM Schwabe D Jacobi G. Acute encephalopathy as a primary manifestation of haemophagocytic lymphohistiocytosis. Dev Med Child Neurol (2001) 43(8):555–8. 10.1017/S0012162201001001 11508922

[50] Murphy C Nanthapisal S Gilmour K Laurent S D’Arco F Hemingway C Progressive neurologic disorder: initial manifestation of hemophagocytic lymphohistiocytosis. Neurology (2016) 86(22):2109–11. 10.1212/wnl.0000000000002729 27164702 PMC4891214

[51] Bryceson YT Pende D Maul-Pavicic A Gilmour KC Ufheil H Vraetz T A prospective evaluation of degranulation assays in the rapid diagnosis of familial hemophagocytic syndromes. Blood (2012) 119(12):2754–63. 10.1182/blood-2011-08-374199 22294731

[52] Mahlaoui N Ouachee-Chardin M de Saint Basile G Neven B Picard C Blanche S Immunotherapy of familial hemophagocytic lymphohistiocytosis with antithymocyte globulins: a single-center retrospective report of 38 patients. Pediatrics (2007) 120(3):e622–8. 10.1542/peds.2006-3164 17698967

[53] Marsh RA Allen CE McClain KL Weinstein JL Kanter J Skiles J Salvage therapy of refractory hemophagocytic lymphohistiocytosis with alemtuzumab. Pediatr Blood Cancer (2013) 60(1):101–9. 10.1002/pbc.24188 22522603 PMC3410971

[54] Maschalidi S Sepulveda FE Garrigue A Fischer A de Saint Basile G. Therapeutic effect of JAK1/2 blockade on the manifestations of hemophagocytic lymphohistiocytosis in mice. Blood (2016) 128(1):60–71. 10.1182/blood-2016-02-700013 27222478

